# Promising Antigens for the New Frontier of Targeted Immunotherapy in Multiple Myeloma

**DOI:** 10.3390/cancers13236136

**Published:** 2021-12-06

**Authors:** Shih-Feng Cho, Lijie Xing, Kenneth C. Anderson, Yu-Tzu Tai

**Affiliations:** 1 Jerome Lipper Multiple Myeloma Center, Department of Medical Oncology, Dana-Farber Cancer Institute, Harvard Medical School, 450 Brookline Avenue, Boston, MA 02215, USA; sifong96@gmail.com (S.-F.C.); Kenneth_anderson@dfci.harvard.edu (K.C.A.); 2 Division of Hematology & Oncology, Department of Internal Medicine, Kaohsiung Medical University Hospital, Kaohsiung Medical University, Kaohsiung 807, Taiwan; 3 Faculty of Medicine, College of Medicine, Kaohsiung Medical University, Kaohsiung 807, Taiwan; 4 Center for Cancer Research, Kaohsiung Medical University, Kaohsiung 807, Taiwan; 5 Department of Hematology, Shandong Provincial Hospital Affiliated to Shandong First Medical University, Jinan 250021, China; xiaopiao423@126.com

**Keywords:** multiple myeloma, MM, immunotherapy, tumor target antigen, immunomodulatory drugs, IMiDs, monoclonal antibody, MoAb, CD38, signaling lymphocyte activation molecule family 7, SLAMF7, B cell maturation antigen, BCMA, bone marrow (BM) microenvironment, orphan G protein-coupled receptor class C group 5 member D, GPRC5D, FcRH5

## Abstract

**Simple Summary:**

Defining the specificity and biological sequalae induced by receptors differentiated expressed in multiple myeloma cells are critical for the development of effective immunotherapies based on monoclonal antibodies. Ongoing studies continue to discover new antigens with superior tumor selectivity and defined function in regulating the pathophysiology of myeloma cells directly or indirectly in the immunosuppressive bone marrow microenvironment. Meanwhile, it is urgent to identify mechanisms of immune resistance and design more potent immunotherapies, alone and/or with best combination partners to further prolong anti-MM immunity.

**Abstract:**

The incorporation of novel agents in recent treatments in multiple myeloma (MM) has improved the clinical outcome of patients. Specifically, the approval of monoclonal antibody (MoAb) against CD38 (daratumumab) and SLAMF7 (elotuzumab) in relapsed and refractory MM (RRMM) represents an important milestone in the development of targeted immunotherapy in MM. These MoAb-based agents significantly induce cytotoxicity of MM cells via multiple effector-dependent mechanisms and can further induce immunomodulation to repair a dysfunctional tumor immune microenvironment. Recently, targeting B cell maturation antigen (BCMA), an even MM-specific antigen, has shown high therapeutic activities by chimeric antigen receptor T cells (CAR T), antibody-drug conjugate (ADC), bispecific T-cell engager (BiTE), as well as bispecific antibody (BiAb), with some already approved for heavily pretreated RRMM patients. New antigens, such as orphan G protein-coupled receptor class C group 5 member D (GPRC5D) and FcRH5, were identified and rapidly moved to ongoing clinical studies. We here summarized the pathobiological function of key MM antigens and the status of the corresponding immunotherapies. The potential challenges and emerging treatment strategies are also discussed.

## 1. Introduction

The development and introduction of the proteasome inhibitor (PI) bortezomib and immunomodulatory drugs (IMiDs), including thalidomide and lenalidomide, has revolutionized the treatment paradigm for multiple myeloma (MM). Second-generation drugs within the same classes, such as carfilzomib and ixazomib (PIs) and pomalidomide (IMiDs), further improve the response rate, survival, and safety profile [1,2,3]. The incorporation of autologous stem cell transplantation in eligible patients has also prolonged survival with more durable disease control [4,5]. However, disease recurrence remains common for most MM patients. Since drug-resistant clones constantly emerge and evolve, leading to a low 5-year overall survival rate in real-world data [6]. The clinical outcomes of patients with relapsed or refractory MM (RRMM) are dismally poor because of the gradually decreased durability of the response to successive lines of anti-MM therapy [7]. It is thus urgent to further devise novel therapies with different mechanisms of action and optimize treatment efficacy to reduce the risk of disease relapse and deepen response durability.

Accumulating studies for the past decades have defined that the bone marrow (BM) microenvironment is essential in supporting MM cell growth, survival, and drug resistance. MM cells are in close contact with surrounding BM accessory cells through bi-directional interactions, including stromal cells (BMSCs) [8], osteoclasts (OCs) [9,10], regulatory T (Treg) or B (Breg) cells [11,12,13], myeloid-derived suppressor cells (MDSCs) [14], tumor-associated macrophages (TAMs) [15], and plasmacytoid dendritic cells [16]. These non-MM cells, in turn, secrete abnormal levels of a variety of cytokines and growth factors in a paracrine fashion to promote pathogenesis of MM, including interleukin-6 (IL-6), IL-10, MIP-1α/β, transforming growth factor-beta (TGFβ), stromal cell-derived factor-1 (SDF-1), and a proliferation-inducing ligand (APRIL) [9,17,18,19]. Furthermore, changes in BM accessory cells and cytokines, either secreted by accessory cells or MM cells via autocrine or paracrine manners, contribute to myeloma cell immune escape, inhibition of myeloma-specific T effector cells, induction of T-cell anergy, and abnormality in Treg cells, resulting in an immunosuppressive microenvironment that impairs immunotherapy [20].

Monoclonal antibodies (MoAbs) binding to selective molecules on the surface of cancer cells have transformed cancer treatment. In principle, these biologically based molecules/proteins induce tumor cell killing mainly dependent on effector functions, including antibody-dependent cellular cytotoxicity (ADCC) via CD16-expressing effector cells (i.e., NK cells, neutrophils, monocytes), complement-dependent cytotoxicity (CDC), and/or antibody-dependent cellular phagocytosis (ADCP) via macrophages. These primary mechanisms of action are distinct from small molecules used in conventional chemotherapies, which directly induce tumor cell apoptosis and are largely independent of immune effector function. The first two therapeutic MoAbs available for RRMM patients are MoAbs targeting CD38 (daratumumab) and SLAMF7 (also named CS1) (elotuzumab), approved by the U.S. Food and Drug Administration (FDA) in 2015 [21,22]. These represent an important breakthrough for effective targeted immune-based therapies in MM. Importantly, results obtained from preclinical and clinical studies of both MoAbs thus far have shown that these first-generation targeting bio-molecules also affect the immunosuppressive non-MM cell components in addition to MM cells [11,12,13,23,24,25]. These findings have inspired many investigations on identifying the patho-immunological roles of various immune regulatory cell subsets and molecules regulating their function using in vitro, ex vivo, and in vivo models. Data from some of these studies have provided rationales to improve the usage of targeting immunotherapeutic reagents with novel approaches. More detailed results will be highlighted in subsequent sections.

Factors to be considered to enhance the effectiveness of current targeted immunotherapies based on MoAb in MM are as follows. First, these agents should more selectively identify and bind to specific cell surface antigens to trigger MM cell cytotoxicity with a minimal off-target impact on normal tissues. Second, in addition to significantly inducing MM cell lysis, these immunotherapeutic molecules could, at the same time, mitigate an immunocompromised BM microenvironment and revert suppressive immune effector cells. Moreover, a combination of different immunotherapeutic platforms with distinct mechanisms of action against target antigens on MM cells as well as key immune stimulatory or inhibitory cells will enhance the strength and reduce the weakness of individual drugs to further prolong the durability of the responses and reduce non-specific toxicity. Currently, immunotherapeutic modalities based on naked MoAbs, chimeric antigen receptor T cells (CAR-T), bispecific antibody (BiAb), bispecific T-cell engager (BiTE), or antibody-drug conjugates (ADC) are the main research areas under preclinical and clinical development [26,27,28]. These agents are mostly designed to target tumor antigens on MM cells and endowed with the ability to restore and further sustain anti-MM effector function in the suppressive BM milieu.

## 2. Pathophysiological Function for Validated MM Target Antigens and Their Related Immunotherapies

**Figure 1:**
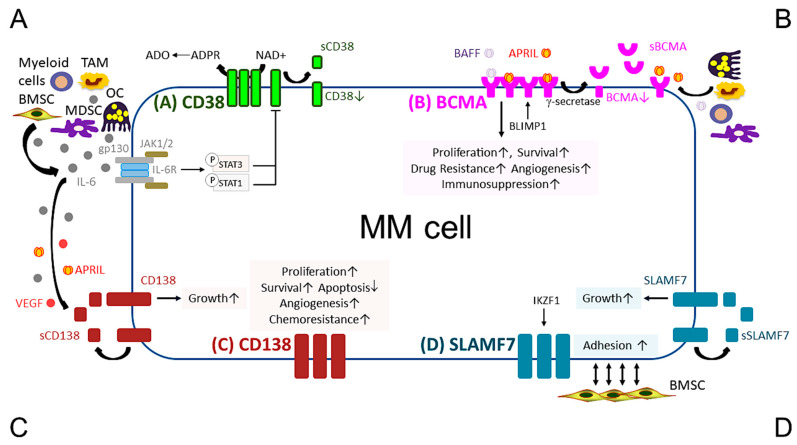
Pathobiological function of validated MM target antigens commonly used in current MM immunotherapies. (**A**) CD38, a receptor and an ectoenzyme, regulates the conversion of NAD+ to ADO, which contributes to the immunocompromised tumor microenvironment. IL-6, an important MM growth, survival, and immunosuppressive cytokine, mainly produced by non-myeloma BM cells (i.e., BMSCs, MDSC, OC, and TAM), binds to IL-6R, which interacts with gp130 to activate the JAK1/2-STAT1/3 signaling pathway and subsequently downregulates CD38 expression in MM cells. (**B**) BCMA is the cognate receptor for APRIL and BAFF, which are present in the BM microenvironment. APRIL, an important plasma cell factor, preferentially binds to BCMA when compared with BAFF, to induce signaling pathways critical to promote survival, proliferation, and drug resistance, as well as immunosuppression of MM cells. BCMA expression is induced by BLIMP1, a key plasma cell transcriptional regulator, and BCMA protein is cleaved by gamma (γ)-secretase, resulting in the soluble form (sBCMA) that is detected in MM patient serum and correlated with disease advancement. (**C**) CD138, the transmembrane heparan sulfate proteoglycan syndecan-1, is overexpressed in malignant plasma cells and its levels are associated with increased proliferation and survival, as well as decreased apoptosis in MM cell lines and patient MM cells. Levels of shed CD138 (sCD138) cleaved by metalloproteinases and heparanase in MM patient serum samples are also linked to disease progression and sCD138 acts locally or distally with various effector molecules (i.e., IL-6, APRIL, and VEGF) to impact tumor progression. (**D**) SLAMF7, also named CS1, promotes adhesion of MM cells to BMSCs. The expression of SLAMF7 is regulated by the transcriptional factor IKZF1 (Ikaros), a MM-related target by lenalidomide and pomalidomide, in MM cells. Soluble SLAMF7 (sSLAMF7) is detected in patient serum and promotes MM cell growth via homophilic interaction with SLAMF7 on MM cells. Among these four MM antigens, BCMA has the most limited expression at the transcript and protein levels in plasma cells but no other normal tissues except a minute subset of plasmacytoid dendritic cell. ADO, adenosine; ADPR, ADP-ribose; APRIL, A proliferation inducing ligand; BAFF, B-cell activating factor; BMSC, bone marrow stromal cell; JAK, Janus kinase; MDSC, myeloid-derived suppressor cell; NAD+, nicotinamide adenine dinucleotide; OC, osteoclast; TAM, tumor-associated macrophages; VEGF, vascular endothelial growth factor.

**Figure 2:**
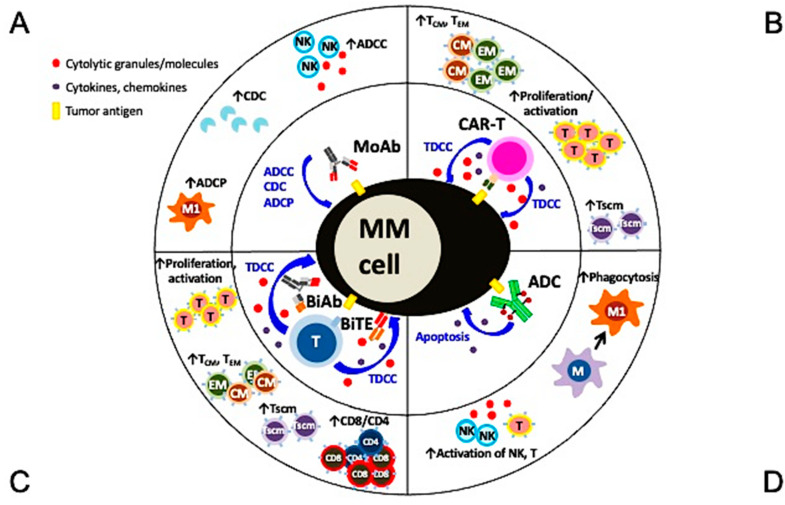
Multiple mechanisms of action and immunomodulatory effects of current targeted immunotherapies in MM. (**A**) Monoclonal antibodies (MoAbs) identify and bind to specific antigens to trigger MM cell lysis via multiple immune-dependent mechanisms, including ADCC, ADCP, and CDC. (**B**) The single chain fragment variant of the CAR-T cell first targets the MM-specific antigen and potent MM cell lysis is induced in a major histocompatibility complex-independent manner followed by increased proliferation and activation of T effector cells, including those with central and effector memory phenotypes. (**C**) BiAb or BiTE, off-shelf products different from autologous CAR-T cells, simultaneously binds to specific tumor antigen on MM cells and CD3 on T cells to trigger potent MM cell killing as well as proliferation of immune effector T cells. T cells with memory phenotypes (Tcm, Tem, Tscm) and the major cytolytic T cells (CD8+ cells) are increased significantly. (**D**) The MoAb portion of antibody drug conjugate (ADC) binds to tumor antigen on MM cell membrane and the ADC is endocytosed followed by the release of potent cytotoxic payloads from lysosomes to directly induce MM cell apoptosis. Payloads used in ADC are typically even more potent than those used in chemotherapies to improve superior specific killing of tumors cells but not the surrounding normal tissues. Depending on the design of the ADC, NK cells and macrophages are directly or indirectly activated to induce ADCC and phagocytosis, respectively, to further augment killing of tumor cells. ADCC, antibody-dependent cellular cytotoxicity (NK cells are the predominant effector cells. Other CD16-expressing effectors including monocytes and neutrophils are also capable to induce ADCC); ADCP, antibody-dependent cellular phagocytosis (macrophage (M), especially with the M1 characteristics, are the key effector cells whereas macrophages with M2 features (TAM in Figure 1) promote tumor growth); BiAb, bispecific antibody engaging with T cells; BiTE, bispecific T cell engager; CDC, complement-dependent cytotoxicity; Tcm, central memory T cell; Tem, effector memory T cell; Tscm, stem cell-like memory T cell.

The anti-MM mechanisms of targeted immunotherapeutic bio-reagents are mainly derived from the MoAbs, which detect and engage with selective proteins on MM cell membrane followed by the induction of NK cell-mediated killing (Figure 1 and Figure 2). These target antigens are chosen based on their differential expression and/or critical roles in growth, survival, and drug resistance in MM cells. The following paragraphs underline the major pathobiological and -immunological characteristics as well as the corresponding targeting immunotherapeutic agents of MM target antigens currently under preclinical and clinical studies.

### 2.1. CD38

CD38, a type II transmembrane glycoprotein, was first identified as a marker of cell activation and proliferation in lymphocytes. It regulates cell migration [29] and receptor-mediated adhesion via interaction with endothelial CD31 or hyaluronic acid [30]. CD38 receptor also exhibits ecto-enzymatic activity, involved in the metabolism of cytoplasmic nicotinamide adenine dinucleotide phosphate (NADP) and extracellular nicotinamide adenine dinucleotide (NAD+) [31]. In addition, CD38 interacts with its substrate, NAD+, to increase production of Ca2+ mobilizing compounds; i.e., cyclic adenosine diphosphate ribose (CADPR), ADP ribose (ADPR), and nicotinic acid adenine dinucleotide phosphate [32,33,34]. ADPR is further converted to adenosine monophosphate (AMP) by CD203a and then adenosine (ADO), which exhibits immunosuppressive activity via a reduction in immune cell activity and induction of differentiation of osteoclast, one of the most immunosuppressive BM accessory cells [35,36,37,38]. Moreover, the malignant plasma cells further use aerobic glycolysis to promote an acidic BM, which together with CD38 highly expressed on their surface induce the generation of AMP and ADO.

In MM cells, CD38 is downregulated by IL-6, the major MM cell growth and survival factor secreted by BMSCs, via activation of IL-6-induced JAK1/2-STAT1/3 signaling pathways [39,40]. Decreased CD38 expression triggered by IL-6 in MM cells is further associated with reduced sensitivity to anti-CD38 MoAb (i.e., daratumumab), providing a new molecular mechanism to support the immunosuppressive function of IL-6 in the BM microenvironment. Since the JAK1/2 inhibitor ruxolitinib blocks IL-6-induced phosphorylation of STAT1 and STAT3, these data also suggest a potential combination daratumumab with JAK1/2 inhibitor to revert daratumumab resistance.

On the other hand, since CD38 is also expressed at various levels in other normal hematopoietic cells, including NK effector cells, daratumumab-induced MM cell lysis is negatively affected due to daratumumab-induced NK cell depletion, as seen in laboratory studies as well as in patients [41,42,43]. New adoptive immunotherapy using ex vivo expanded human primary NK cells with or without CD38 knockout was recently proposed to boost daratumumab activity in MM [42,43]. In contrast, when compared with immune effector T cells, immune inhibitory Treg and Breg cells express elevated CD38 levels, as high as MM cells, and thereby are preferentially eliminated by CD38 targeting MoAbs and T cell expansion is promoted [11,12,44]. This additional function on targeting key immune suppressive cell subsets further support the therapeutic efficacy of anti-CD38 MoAbs in MM.

The incorporation of daratumumab into current anti-MM treatment regimens has significantly prolonged overall survival in RR as well as ND MM patients [22,45,46,47,48]. In 2019, daratumumab is the first MoAb, when used in combination, approved for treatment for NDMM patients who may still exhibit functional immune cells in the less defective immune BM milieu. The second anti-CD38 MoAb, isatuximab, recognizes the non-overlapping CD38 epitope of daratumumab and has shown convincing clinical activity, leading to its approval when combined with pomalidomide and dexamethasone in RRMM in 2020 [49].

Besides the induction of effector-mediated MM cell lysis in a CD38-dependent manner, both daratumumab and isatuximab eliminate high CD38-expressing immune inhibitory cell subsets (i.e., Treg, Breg) in MM patients, a supplementary mechanism to increase immune effector cell number and function [11,12,13]. Compared with NK effector cells, T effector cells express low levels of CD38. A BiTE, AMG 424, with a lower CD38-binding affinity together with CD3 binding to T cells, was recently developed and its preclinical activity was evaluated using in vitro and in vivo models of human MM [50]. This next generation CD38-targeting molecule potently induces a cytotoxic T cell response to kill MM cells via cytolytic cytokine production (i.e., interferon-gamma (IFNγ), granzyme B (GZMB), perforin (PRF1)), with minimal toxicity on T effector cells. Furthermore, anti-CD38 CAR-T cells were reported and induced lysis of CD38+ MM cells with mild effects on other CD3+ T cells [51].

### 2.2. SLAMF7 (CS1)

SLAMF7 is a member of the immunoglobulin gene superfamily (signaling lymphocyte activation molecule family) and associated with cytotoxic effector, humoral and auto-immunity, and cell survival/adhesion, as well as lymphocyte development [52,53]. SLAMF7 is also expressed on the surface of certain subsets of immune cells, including NK cells, cytotoxic T lymphocytes (CD8+ cells), B lymphocytes, and mature dendritic cells. SLAMF7 itself can serve as a receptor of NK cell and it is a self-ligand that exhibits a homophilic interaction to augment NK cell-mediated cytotoxicity [54]. Moreover, SLAMF7 on the surface of B cells is upregulated during B cell activation to promote proliferation of naive and memory B cells and cytokine production [55].

SLAMF7 is highly expressed on patient MM cells from ND to RRMM patients, independent of the cytogenetic risk classification [24,56]. Soluble SLAMF7 (sSLAMF7) is detected in MM patients rather than normal individuals [24] and could promote MM cell growth via activating the SHP-2 and ERK signaling pathways via homophilic interaction [57]. Targeting IKZF1 (Ikaros 1), a critical transcriptional activator of SLAMF7, by IMiDs downregulates SLAMF7 expression and ameliorated the response of MM cells to sSLAMF7. MM cells with t(4;14) translocations (15% of all MM cases) have higher SLAMF7 expression, associated with MMSET overexpression [58]. SLAMF7 knockdown by its shRNA inhibits colony formation and induces cell cycle arrest followed by apoptosis of t(4;14) plasma cells, indicating elevated SLAMF7 expression in promoting the growth of MM cells. Furthermore, SLAMF7 promotes the adhesion of plasma cells to BM stromal cells to support survival and proliferation of MM cells in the BM; conversely, an antagonistic anti-SLAMF7 MoAb prevents MM cells from adhesion to BMSCs and induces ADCC to lyse MM cells [24,59]. Most recently, SLAMF7 was found highly expressed on immunosuppressive CD8+CD28-CD57+ Tregs in MM patients and these cells could be eliminated using anti-SLAMF7 MoAb elotuzumab [25].

Elotuzumab, the first-in-class humanized immunoglobulin G1 anti-SLAMF7 MoAb, exhibits immunomodulatory effects on NK cells via activation of SLAMF7/EAT2 signaling without negative impacts on NK cell number and survival [24,56,60]. Combination of elotuzumab with IMiDs (lenalidomide, pomalidomide) with low-dose dexamethasone significantly improved the clinical outcome of RRMM patients in the ELOQUENT-2 trial [21,61], leading to its approval for the treatment in RRMM. SLAMF7-targeting agents using ADC, CAR-T, or BiAb are under preclinical and/or clinical investigations.

### 2.3. CD138 (Syndecan-1)

CD138 (syndecan 1), a member of the syndecan family of type I transmembrane proteoglycan, has been commonly used as a prognostic marker in MM, since its expression level is elevated in malignant versus normal plasma cells [62]. CD138 modulates various biological processes, including proliferation [63], adhesion [64], migration [65], endocytosis [66], macropinocytosis [67], immunomodulation [68], and regulation of heparan sulfate proteoglycans [69]. Increased CD38 expression promotes proliferation and survival of MM cells, as well as angiogenesis and IL-6 receptor sensitivity in MM cells [70,71]. IL-6-induced growth and survival signaling cascades upon binding to IL-6R is further augmented in MM cells overexpressing CD138, indicating cross-talks between CD138 and IL6R in the progression of MM. Importantly, high CD138 expression is linked to enhanced malignant plasma cell growth and disease burden in patients. Since CD138 is cleaved by metalloproteinases and heparanase, soluble CD138 (sCD138) is detected in patient serum samples and its levels are associated with the prognosis of MM, with shorter survival in patients with higher levels [72]. Significantly, shedding of CD138 (sCD138) from MM cells stimulates myeloma cell growth by positive regulation and interaction with other MM-promoting factors (i.e., IL-6, vascular endothelial growth factor (VEGF), APRIL) in the BM microenvironment [62,70,73].

Besides plasma cells, CD138 is expressed in other normal and malignant human tissues at various levels, including normal squamous epithelial, goblet, and columnar cells in the gastrointestinal tract, as well as tumor cells, including squamous cell carcinoma and adenocarcinoma [67,74,75]. As reported in previous clinical studies testing the first CD138-targeting ADC, BT062, described below, high expression of CD138 on epithelial cells was associated with increased risk of treatment toxicity (such as mucositis or diarrhea) [76].

BT062/Indatuximab ravtansine, an ADC with cytotoxic maytansinoid DM4, was the first CD138-targeting drug tested in an MM clinical trial [76,77]. Most of the adverse events were mild (grade 1 or 2), with diarrhea and fatigue the most common. The response rate of monotherapy or combination therapy (with lenalidomide or pomalidomide and dexamethasone) in RRMM patients were 14.7% and 77%, respectively [76,77]. Besides ADC, CD138-targeting therapy under clinical and preclinical investigation include CAR-NK and CAR-T cell therapy [78,79,80]. A case report demonstrated a patient with extensive extramedullary MM involvement receiving anti-CD138 CAR-T cell infusion (total: 1.5 × 10^8^) after a conditioning regimen of cyclophosphamide and fludarabine. The patient experienced grade 2 cytokine release syndrome (CRS) and received anti-IL-6R MoAb tocilizumab treatment with clinical partial response (PR) [81].

Recently, a bispecific antibody and CAR-T cell based on a new anti-CD138 MoAb showed significant anti-MM activity and an immunomodulatory effect in preclinical studies [78]. A novel naked MoAb VIS832 was recently made, showing enhanced membrane CD138-binding affinity compared to the MoAb portion of BT062 [82]. VIS832, as a naked IgG1 MoAb, potently induced ADCC and ADCP against MM cells, including resistant cell lines or patient MM cells, without directly impacting MM cells. No toxicity was seen in NK cells treated with VIS832, confirming the absence of CD138 expression on immune effector cells. Anti-MM activity of VIS832 in the in vitro and in vivo models was further augmented when combining with lenalidomide or bortezomib in the preclinical study. These data provide a clinical rationale to test this new anti-CD138 MoAb, alone or in combination with current standard-of-care anti-MM drugs in MM.

### 2.4. B-Cell Maturation Antigen (BCMA)

BCMA, also called tumor necrosis factor receptor superfamily member 17 (TNFRS17) or CD269, is a type III transmembrane protein with extracellular domains rich in cysteine without a signal peptide. BCMA, closely related to B-cell activation factor receptor (BAFF-R), and transmembrane activator and calcium modulator and cyclophilin ligand interactor (TACI), regulates B cell proliferation and survival, as well as maturation and differentiation into plasma cells [83,84,85]. These three functionally related receptors bind to their cognate ligands, BAFF and/or APRIL, with different affinities, to support long-term survival of B cells at different stages of development. Specifically, BCMA, but not BAFF-R or TACI, is crucial for the long-term survival of plasma cells, but not overall B cell homeostasis [85]. During the differentiation of B cells into plasma cells, the expression of BCMA is induced from late memory cell, while BAFF-R is concomitantly extinguished. BCMA expression is regulated by B-lymphocyte-induced maturation protein 1 (BLIMP1), an important plasma cell transcriptional factor [86]. Under normal physiological conditions, the membrane BCMA is cleaved by gamma-secretase to form the soluble BCMA (sBCMA) [87]. Serum levels of sBCMA are significantly higher in MM patients than healthy individuals and associated with immune deficiency in the tumor microenvironment [88,89]. Elevated sBCMA levels are positively linked to increased tumor burden and poorer overall or progression-free survival [89]. Furthermore, the post-treatment levels of sBCMA could be used as a predictive marker for treatment response [90,91,92].

As in the case for CD38, SLAMF7, and CD138, expression of BCMA is increased in patient MM cells. The evaluation of patient samples from various cohorts further confirmed that expression of BCMA at the transcript and protein level is more restrictively expressed on plasma cells but no other normal tissues, when compared with the above MM antigens included in the previous paragraphs [93,94,95]. Among other normal tissues, BCMA transcript and protein are only weakly detected on plasmacytoid dendritic cells, a minute BM cell subset that promote MM cell growth, survival, and resistance to anti-MM drugs [95]. Patients with higher BCMA expression from their CD138+ plasma cells also have elevated BCMA levels in autologous plasmacytoid dendritic cells, further supporting the patho-biological role of BCMA in MM. In addition, BCMA is co-immunoprecipitated with interferon regulatory factor 4, a major transcription factor mediating survival of MM cells [96]. Overexpression of BCMA directly augments MM cell growth and survival via induction of protein kinase B (AKT), MAPK, and nuclear factor (NF)-κB signaling cascades, followed by upregulation of the gene expression of molecules critical in growth and anti-apoptosis [18,19,84]. It further enhances expression of genes related to activation of OCs, adhesion, angiogenesis, and metastasis, as well as development of immunosuppressive characters, including programmed death ligand 1 (PD-L1), TGFβ, and IL-10. Moreover, overexpression of BCMA in the MM cell line expressing low BCMA levels induces early onset and increased volume of xenografted tumors with increased CD31/microvessel density and VEGF in a murine model of RPMI8226 MM cells, confirming its tumor-promoting effects in vivo.

As a critical plasma cell receptor, BCMA binds to its cognate ligands BAFF or APRIL to activate AKT, ERK1/2, and NFκB pathways in MM cells [18,97]. These two ligands are mainly secreted by non-MM cells in the BM and with differential influences on the biology of MM cells. First, BAFF binds to BAFF-R, BCMA, and TACI, to promote adhesion of MM cell to BM stromal cells, but with a significantly higher selectivity (~100-fold) to BAFF-R, which is hardly detectable in MM cells [97,98]. In contrast, APRIL cannot bind to BAFF-R and preferentially binds to BCMA or TACI, the latter of which is less frequently expressed when compared with the former in MM cells. Specifically, APRIL preferably binds to BCMA with much higher affinity than BAFF [83,99] and is predominantly produced by myeloid cells, macrophages, OC precursor cells, and OCs which play central pathophysiological roles in MM-induced bone lesions. All these data indicate that APRIL may be a more significant factor than BAFF in the development and progression of MM. Importantly, APRIL binding to BCMA triggers multiple signaling pathways to further promote drug resistance of MM cells and the progression of immunosuppressive BM milieu via induction of the key downstream anti-apoptotic genes (Mcl-1, Bcl-2/Bcl-xL) and immune regulatory genes (IL-10, PD-L1, VEGF, TGF-β) in MM cells [18,19]. Moreover, APRIL directly impacts Treg cells that express no BCMA, to promote an immunosuppressive BM microenvironment via binding to TACI whose expression levels are significantly correlated with upregulated Treg markers, including Foxp3 and CTLA-4 [44]. Besides TACI-dependent induction of cell cycle progression and anti-apoptosis genes, APRIL specifically augments expression of Foxp3, IL-10, TGFβ1, and PD-L1 in Tregs to further augment Treg-inhibited conventional T cells proliferation and cytolytic function. APRIL also enhances IL-10-producing Breg cells via TACI in the BM of MM patients. All these results strongly support targeting APRIL/BCMA and APRIL/TACI systems for novel MM immunotherapies.

The first therapeutic anti-BCMA MoAb J6M0 was selected based on its significant blocking activity induced by BCMA binding to BAFF or APRIL in the single to sub-digit nanomolar range [95]. J6M0 was subsequently conjugated via non-cleavable linker with a novel anti-tubulin drug monomethyl auristatin F (MMAF) (GSK2857916, now Belantamab mafodotin). GSK2857916 showed more selective and potent anti-MM killing than its monomethyl auristatin E (MMAE) ADC homolog in the preclinical study, thereby moving forward to the first clinical trial of BCMA-targeting ADC in MM [83,95]. GSK2857916 directly induces MM cell apoptosis and simultaneously stimulates ADCC and ADCP via NK and macrophages, respectively. Since 2015 [83], there have been overwhelming advances of new BCMA-based immunotherapeutic agents, including ADC delivering novel potent drugs with different mechanisms to induce MM cell apoptosis, CAR-T, or NK cells, as well as BiTEs and BiAbs engaging T or NK effector cells. Impressively, preclinical studies of these innovative agents utilizing various in vitro and in vivo models consistently demonstrate robust anti-MM cytotoxicity with some having been further translated into significant clinical activities [93,94,95,100,101,102]. All early phase clinical studies in small patient cohorts showed a promising high response rate and durable disease control in heavily pretreated RRMM patients [27,103,104]. Several anti-BCMA agents have completed or entered phase 2 or 3 studies [27]. In 2020, belantamab mafodotin and idecabtagene vicleucel (ide-cel; formerly bb2121) were the first anti-BCMA ADC and CAR-T therapy, respectively, approved by FDA as a single agent for heavily pretreated RRMM patients. Most recently, teclistamab, a BiAb targeting BCMA and CD3 (a Humanized BCMA CD3 DuoBody^®^ Antibody), received FDA breakthrough therapy designations for RRMM [105]. Today, BCMA targeting immunotherapy is the first targeted therapy inducing an impressive clinical response as monotherapy in heavily pretreated MM patients who have no more treatment options left.

## 3. Other MM Tumor Antigens for Emerging Targeted Immunotherapy

The success of recent CAR-T, BiTE, and ADC, based on BCMA-targeting therapies, has quickly stimulated further development of immunotherapy targeting other novel antigens. Data of clinical studies further confirm that exclusive and high expression of tumor antigens on cancer cell is a key factor for new target selection to maximize the potency while minimizing the risk of off-target toxicity. Orphan G protein-coupled receptor, class C group 5 member D (GPRC5D), is a newly identified MM antigen that is highly expressed on MM cells in the BM but not normal tissue, although weakly expressed in hair follicles [106]. GPRC5D CAR-T exhibiting potent anti-MM activity in a preclinical study has led to ongoing clinical studies. BiAbs targeting GPRC5D and CD3 (talquetamab/JNJ-64407564 and GPRC5D TRAB) have also shown potent T-cell-mediated killing of GPRC5D+ MM cells and proliferation/activation of T cells in the preclinical and ongoing clinical studies [107,108]. Furthermore, the expression level of GPRC5D on MM cells and the BM microenvironment-related factors contribute to a different degree of responses to JNJ-7564 [109]. The early phase clinical trials of talquetamab (JNJ-64407564), as a monotherapy (NCT03399799) or combined with other anti-MM agents (NCT04108195), are ongoing, with already significant clinical activity.

Another potential antigen, integrin β7 (ITGB7), is associated with adhesion of MM cells to extra-cellular matrix elements, migration, invasion, and drug resistance [110]. In the in vitro study, novel ITGB7 targeting MMG49-derived CAR T cells showed specific MM cell lysis without damaging normal hematopoietic cells [111].

Natural Killer Group 2D (NKG2D) ligand, expressed on about 80% of MM cells, can bind to NKG2D on natural killer cells, leading to immune escape and tumor growth [112,113]. A BiAb targeting NKG2D and CS1 showed significant immune synapse between CS1+ MM cells and NKG2D+ immune cells, leading to effective MM lysis [114]. NKG2D-CAR T cells was also evaluated in a clinical trial, which showed good safety, but no objective response was observed (NCT02203825) [115].

CD229, a member of the SLAM family, is highly and homogenously expressed on MM cells and myeloma precursors, but not on other normal tissues [116,117]. A preclinical study showed anti-CD229 CAR-T cells exhibited potent in vitro and in vivo activity to against MM and MM-propagating cells, with minimal damage to normal T cells [118].

FcRH5 (or FcRL5, CD307), a membrane protein highly expressed on mature B cells and plasma cells, was also evaluated in MM treatment [119]. A BiAb, cevostamab (BFCR4350A), targeting FcRH5 and CD3, was constructed and showed significant in vitro and in vivo anti-MM activity, as well as T cell activation/proliferation in a preclinical study [120]. The phase 1 trial evaluating the safety and efficacy of cevostamab in RRMM is ongoing (NCT03275103).

Besides the above MM antigens, CD44v6 [121], immunoglobulin light chain [122], and CD19 [123] was also evaluated previously. These antigens showed a significant in vitro and in vitro anti-MM effect, or promising results as multi-target combination immunotherapy [124].

## 4. Potential Challenges and Strategies

Multiple factors are associated with lower treatment efficacy and immune resistance, frequently seen in heavily pretreated patients. First, downregulation of tumor antigen reduces the binding affinity of target agents, resulting in lower tumor killing. Low expression of CD38, together with increased membrane expression of CD55 and CD59, two important immune inhibitory molecules in complement-mediated tumor cell lysis, were associated with treatment resistance to daratumumab [41]. Second, increased shedding of tumor antigens into a soluble form in the serum could act as a decoy, which naturalizes the immune-targeted agents and negatively affects the pharmacokinetic profile and treatment response. High serum levels of soluble SLAMF7 have been associated with a poorer response to elotuzumab and shorter survival [125]. Likewise, sBCMA and sCD38 could reduce the anti-MM activity of anti-BCMA BiTE [102] or daratumumab [126], respectively. Another mechanism contributing to resistance is antigen escape (or loss), which was recently reported in plasma cells in relapsed MM patients with a very low level or loss of BCMA expression, mostly in anti-BCMA CAR-T cell trials [90,91,127]. These low or no antigen-expressing MM cells may be selected out and proliferate after normal or high tumor antigen-expressing cells were eradicated by potent immunotherapy, leading to disease progression. A study using a mouse model revealed that both CD28- and 4-1BB-based CARs were able to induce reversible antigen loss by trogocytosis, which promote the transfer of target antigens to T cells [128]. Most recently, two correlative studies using a sequencing technique to analyze patient samples sequentially collected revealed that biallelic loss or homozygous gene deletion of BCMA play a critical role in antigen escape [129,130]. In MM patients who have not been previously treated with BCMA-targeting therapies, BCMA loss or monosomy 16 was observed in 22% (37/168) of them. Moreover, a significantly higher percentage was noted in patients with hyperdiploid cytogenetics (84.8%, 28/33) [130]. Furthermore, the immunocompromised BM microenvironment is aggravated by MM cell-induced abnormal increased Treg cells, MDSCs, OCs, and/or Breg cells, as well as upregulation of their secretory immunosuppressive cytokines, i.e., IL-6, IL-10, and TGFβ. Enhanced PD-L1 expression on MM cells and other BM accessory cells engages PD-1 on activated effector T cells to suppress their proliferation and production of cytolytic cytokines, leading to a functionally exhausted state and further apoptosis [9,11,19,131,132]. In addition to PD-1, aberrant enhanced or prolonged expression of other immune checkpoint molecules, i.e., TIM-3, CTLA-4, and LAG-3, in NK and T effector cells further inhibit their number and effector function upon binding to their cognate ligands upregulated in MM cells [133].

Several strategies are and/or have been under preclinical or clinical investigations. For decreased MM antigen expression, selective therapeutic agents are shown to enhance target expression on the MM cell surface. In preclinical studies, all-trans retinoic acid, histone deacetylase (HDAC) inhibitors, and JAK/STAT3 inhibitors were reported to induce CD38 expression, which was associated with enhanced CD38 targeting by daratumumab [134,135,136,137]. Since CD38 is a NAD+-degrading enzyme producing ADO, which is immunosuppressive, as mentioned in the above section, the depletion of NAD+ may decrease generation of ADO and allow more CD38 targeting by anti-CD38-based immunotherapies [35]. Most recently, a new DNA-damaging drug specifically delivered to MM cells through BCMA targeting was reported to increase the CD38 levels and further overcome daratumumab insensitivity in MM cell lines and patient MM cells [40]. For BCMA-based immunotherapies, gamma-secretase inhibitors could reduce the shedding of BCMA from MM cells, associated with increased levels of cell membrane BCMA and MM cell killing [87,138]. The preliminary result is promising in an early phase trial evaluating the combination effect of anti-BCMA CAR-T cell therapy and gamma-secretase inhibitor in RRMM patients (NCT03502577) [139].

Modification of the MoAb structure via protein engineering to increase the binding affinity to the membranous form of the target antigens could significantly augment MM cell targeting, as reported for a novel anti-BCMA ADC [140] and anti-CD138 VIS832 [82]. For antigen loss identified in patients refractory to BCMA CAR T treatment, preclinical studies have demonstrated that targeting the new antigen GPRC5D using CAR-T or BiTE could still effectively induce lysis of the BCMA knockout MM cell lines [106,108]. A preclinical study evaluating bispecific CAR-T cells simultaneously targeting both BCMA and SLAMF7 on MM cells showed promising results to prevent antigen escape and better control MM cells heterogeneously expressing these antigens [141]. Significantly, BCMA/CS1 bispecific CAR-T cells induce superior CAR expression and function compared to T cells co-expressing individual BCMA and CS1 CARs. Furthermore, combination of an antagonistic anti-PD-1 MoAb with BCMA/CS1 bispecific CAR-T cells accelerates the rate of initial tumor clearance in a murine model, while CAR-T cell treatment alone achieves durable tumor-free host survival even upon tumor re-challenge.

**Figure 3:**
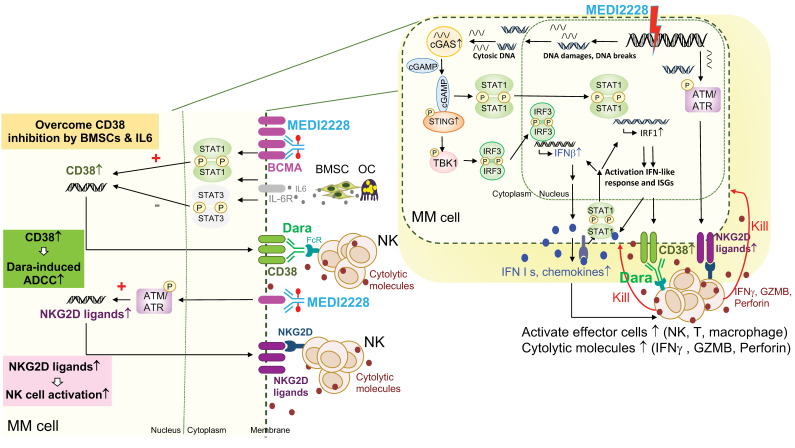
Molecular and cellular mechanisms for enhanced MM cell killing by simultaneously targeting BCMA and CD38. Combination BCMA- and CD38-directed therapies hold great promises to improve the potency and durability of current targeted immunotherapies in MM. For instance, as shown here, the additional immunomodulatory effects and molecular mechanisms of the BCMA-specific ADC, MEDI2228, could augment NK cell killing of drug-resistant MM cells in the presence or absence of daratumumab (dara) via enhanced MM cell recognition and destruction by effector cells. Although only showing NK cells here, T and macrophages also will be activated by inflammatory cytokines (i.e., IFN I molecules) and chemokines secreted from MM cells, to further boost the anti-MM immune function of this ADC. Significantly, MEDI2228 activates DNA-damage-related ATM/ATR-CHK1/2 and cGAS/STING/TBK1/IRF3, as well as STAT1/IRF1 signaling cascades followed by the induction of downstream type I IFN and IFN-stimulated genes in MM cells (right panel). Cell membrane expression of CD38, an IFN-associated gene, and NKG2D ligands (MICA/B, ULBP2/3/5) are elevated in MEDI2228-treated MM cells, thereby further restoring CD38 targeting by Dara.

A novel anti-BCMA pyrrolobenzodiazepine (PBD) ADC, MEDI2228, preferentially binds to membrane BCMA [142] and further induces DNA damage-induced ATM/ATR-CHK1/2, cGAS-STING-TBK1-IRF3, and STAT1-IRF1-signaling cascades to activate IFN-related molecules [40,143] (Figure 3). Significantly, MEDI2228 upregulates expression of CD38 and multiple NKG2D ligands on the MM cell membrane in vitro and in a xenograft murine model of human MM. It overcomes CD38 downregulation triggered by IL6 via activation of STAT1/IRF1 and further restores daratumumab-induced ADCC against resistant MM cell lines and patient MM cells. Unlike daratumumab, which depletes NK cells due to CD38 expression, MEDI2228 has no impact on NK cells. Upregulation of NKG2D ligands as “eat me” signals, including MICA/B and ULBP2/3/5, further augment binding of MEDI2228-treated MM cells to the NKG2D receptor on NK cells, resulting in increased NK immune surveillance as well as enhanced daratumumab-induced MM cell killing in in vitro and in vivo preclinical study models. Importantly, combination daratumumab and MEDI2228 treatment led to all mice bearing MM1S tumors becoming tumor-free with 100% survival. These results further support clinical rationales to test the combination CD38- and BCMA-targeting immunotherapies to achieve effective and durable anti-MM activity. Such combination clinical studies are ongoing based on belantamab mafodotin, the first approved BCMA ADC, in MM, including NCT04246047 with daratumumab and NCT04643002 with isatuximab.

Many ongoing investigations (mainly CAR-T cell therapy) are to evaluate dual-targeting strategies with various combinations based on BCMA, CD19, CD138, and CS1 (SLAMF7), together or in sequence (Table 1), and some already showed improved clinical activity in early phase clinical studies [27,124]. Regarding treatment approaches to augment anti-MM activity of a single targeted immunotherapeutic agent or to overcome an immunosuppressive BM microenvironment, a combination of different anti-MM agents with distinct mechanisms of action remains very attractive strategies. For example, IMiDs, which enhance immune effector cell function and block immunosuppressive BM accessory cells, have been commonly combined with many current anti-MM treatments [144,145]. A preclinical study has shown that lenalidomide enhances cytotoxic effect anti-CS1 CAR-T cells in in vitro and in vivo MM models [146]. Recent studies also demonstrated that lenalidomide or pomalidomide, as well as an PD-L1 inhibitor, further augment T-cell mediated MM cell lysis and immune modulatory function of half-life-extended anti-BCMA BiTE AMG 701 [100,101]. IMiDs further upregulated AMG 701-induced patient T-cell differentiation toward memory phenotypes, associated with increased CD8/CD4 ratios and stem-like T cells (Tscm), as well as decreased IL-10+ T and Treg cells that downregulate T effector cells. Importantly, the combination of AMG 701 with lenalidomide further prolonged host survival following sustained inhibition of MM cell growth in SCID mice reconstituted with human T cells. As previously mentioned, bortezomib or daratumumab also enhance the anti-MM effect of an anti-BCMA ADC MEDI2228 in preclinical studies [40,143]. Furthermore, VIS832 with superior CD138-binding affinity, significantly augments MM cell killing in vitro and in vivo when combined with lenalidomide or bortezomib [82].

## 5. Perspectives

**Figure 4:**
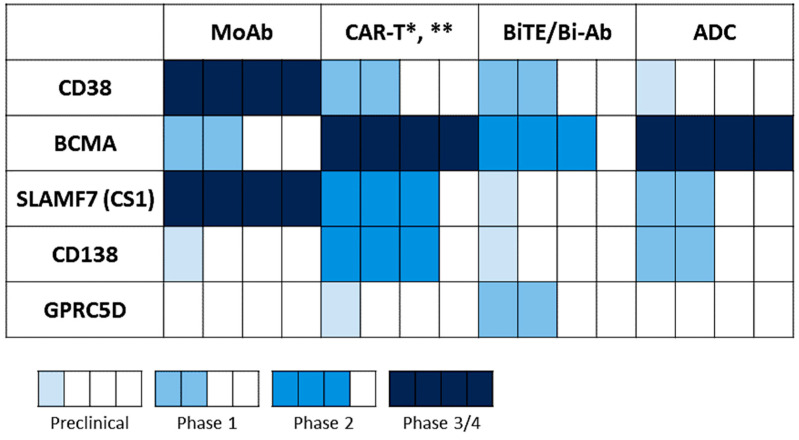
Statuses of various targeting immunotherapeutic agents under development in MM. Statuses of different immunotherapeutic formats based on the indicated MM target antigens were summarized from the ClinicalTrials.gov website (data cut-off: 14 October 2021). * CAR T therapy has moved to allogeneic strategies, including BCMA CAR T (NCT05000450 (ALLO-605), NCT04093596 (ALLO-647), NCT04960579 (P-BCMA-ALLO1)) and CS1/SLAMF7 CAR T (NCT04142619 (UCARTCS1A)). ** The BCMA CAR-NK trials are ongoing, including NCT03940833 in phase 1/2 and NCT05008536 in phase 1.

Since the approval of daratumumab and elotuzumab in 2015, more targeted approaches based on the highlighted antigens above have been generated and investigated in different phases of clinical development, as monotherapy and/or in combination (Figure 4, Table 1). To date, BCMA-based immunotherapy represents the most promising targeted approach, as many clinical studies of different immunotherapeutic formats have showed impressive overall response rates (>70–90%) with prolonged disease control duration, achieving minimal residual disease (MRD) negativity in RRMM patients [27,104,147]. However, a significant portion of patients still suffered from progression or relapse of disease; thus, further optimization of the current treatment approaches, using various immunotherapeutic forms targeting single, dual, or multiple antigens and/or in combination, are urgently needed to prevent disease recurrence and deepen treatment responses.

For the anti-CD38 treatment, the MoAbs recognizing the distinct epitope of CD38, such as isatuximab and MOR202, have been under several clinical investigations, with isatuximab approved in August 2021. Like daratumumab, isatuximab is characterized by multiple effector-dependent anti-MM mechanisms (ADCC, ADCP, and CDC). In contrast, isatuximab, in the absence of effector cells, can further induce apoptosis of MM, Treg, and Breg cells, expressing even higher CD38 levels, while daratumumab cannot [148,149]. A new humanized IgG1 anti-CD38 MoAb, mezagitamab (TAK 079), also showed robust anti-MM activity in RRMM samples and immunomodulatory effects, including activation of NK and T cells, as well as suppression of immune inhibitory cells [150]. The early result of a phase 1 study (NCT03439280) is encouraging, with overall response rate of 31% and a good safety profile [151]. Another novel anti-CD38 ADC, TAK-169, using deimmunized Shiga-like toxin A subunit as the payload, also demonstrated a remarkable in vitro anti-MM effect [152]. TAK-573, a humanized, anti-CD38, IgG4 MoAb genetically fused to two attenuated IFN alpha-2b molecules, showed further increased cytotoxic potential of CD8 T cells via modulation of the IFN-α receptor pathway after treatment in an early phase clinical study [153]. Strategies to modulate the glycosylation of the Fc portion (glyco-engineering) may enhance the affinity of the antibody for FcγRs, thereby resulting in more potent direct and immune effector cell-mediated cytotoxicity. A novel, hexamerization-enhanced human IgG1 anti-CD38 antibody (GEN3014) with an E430G mutation to enhance intermolecular Fc–Fc interactions showed potent CDC activity and ADCC in a preclinical study [154]; an early phase clinical study was recently initiated (NCT04824794). Furthermore, a preclinical study evaluated a BiAb targeting CD38 and CD59 showed a significant increased CDC activity, which was mediated by simultaneously binding to CD38 and neutralization of CD59, which is associated with daratumumab resistance [155].

Next-generation modalities targeting SLAMF7 are also under development. ABBV-838, the first anti-SLAMF7/CS1 ADC, is characterized by cytotoxic MMAE as the payload and an enzymatic cleavable valine-citrulline linker, despite the first-in-human clinical trial of this ADC showing a low response rate in RRMM patients [156]. Other anti-CS1 agents, such as CAR-T cell therapy or anti-CS1/NKG2D BiAb, also showed significant in vitro and in vivo anti-MM activity in preclinical studies [114,146]. Anti-CS1 CAR-T cell therapy, including allogeneic CAR-T cells (NCT04142619), is currently under clinical investigation.

For further improvement of anti-BCMA immunotherapy, the novel ADC MEDI2228 preferentially binds to membrane BCMA showed significant clinical activity in RRMM patients, with an overall response rate of 61% and no keratopathy reported in an early phase clinical study [157]. MEDI2228 can be combined with bortezomib and further upregulates CD38 in MM cells and increased immune surveillance via NK cells to overcome daratumumab resistance [40]. Regarding BiTE therapy, a novel half-life-extended anti-BCMA (AMG 701), with a better pharmacodynamic profile, has demonstrated remarkable anti-MM activity in vitro and in vivo [100,101]. The early phase clinical trial investigating weekly dosing of AMG 701 showed promising results and a good safety profile in heavily pretreated RRMM patients (NCT03287908) [147]. For better outcome of anti-BCMA CAR-T cell therapy, structural or protocol modification are also under evaluation. First, a fully humanized and smaller size scFv may reduce the immune response against murine scFv, to improve post-infusion persistence and overcome treatment failure [158]. For example, CART-ddBCMA, a CAR-T cell characterized by utilization of 73 amino acids as the binding domain rather than conventional scFv to reduce immunogenicity, also showed a high response and good safety profile in RRMM patients with high tumor burden (NCT04155749) [159]. Second, a recent study evaluating the pre- and post-infusion sample in the bb21217 trial revealed that the presence of early memory-like T cells in peripheral blood mononuclear cells may be linked to high peak expansion and better disease control, but highly differentiated or senescent T cells exhibited a negative effect [160]. For better treatment safety, novel mRNA-generated CAR-T cells (Descartes-08), to limit excessive proliferation to reduce the risk of cytokine-releasing syndrome (CRS), has shown robust in vitro and in vivo anti-MM activity [161]. The early phase clinical study is ongoing (NCT03448978). In addition to autologous CAR-T cells, the clinical investigation of allogeneic CAR-T cell ALLO-715 is ongoing to test whether off-shelf CAR-T products are feasible to reduce the cost, time, and success of CAR-T generation (NCT04093596). Moreover, NK-based cellular therapy may also exhibit potent anti-MM killing but with a lower risk of fetal CRS than CAR-T cell treatment, since NK cells tend to survive shorter than T cells after infusion (NCT03940833, NCT05008536).

Several strategies to further optimize anti-MM immune targeted therapy are also under exploration. In the BiAb approach, novel treatment approaches, such as BiAb-armed T cells, are emerging in MM treatment. To avoid antigen-loss-related treatment failure, bicistronic CAR, bivalent “tandem CARs”, or CARs with three specificities are under development [162]. Clinical studies of the above novel concept using these validated MM antigens are expected.

Moreover, the first off-the-shelf multiplexed engineered NK cell therapy (FT538) generated from a clonal master engineered-induced pluripotent stem cell is characterized with CD38 knock-out to avoid damage from anti-CD38 MoAb. An early phase of FT538 combined with daratumumab or elotuzumab in RRMM patients is ongoing (NCT04614636) [163].

Besides the therapeutic role, the exclusive expression of MM-specific antigens on MM cells provides a rationale to develop diagnostic tools. ImmunoPET imaging, which utilized the conjugation of deferoxamine-p-benzyl-isothiocyanate to elotuzumab or daratumumab to enable radiolabeling of zirconium-89, showed the optimal detection ability of MM cells in preclinical studies [164,165]. The clinical study to explore the diagnostic role of this novel technique is ongoing (NCT04814615). The combination of these state-of-the-art imaging tools with minimal residual disease assessment may provide more valuable clinical information.

## 6. Conclusions

Landscapes of targeted treatments using immunotherapeutic approaches is continuously evolving, with increasing numbers of novel effective agents granted for approval. Research areas continue to be focused on identification of more specific new target antigens, inhibition of shedding and/or escape/loss of target antigens, modulation of binding affinity to validated and/or new target antigens on MM cells and immune molecules on immune effector or suppressive cells using various immunotherapeutic platforms, and blockage of immune inhibitory cytokines, as well as reprogramming the tumor immune microenvironment in favor of persistent anti-MM immunity. Combinations within individual targeted reagents and/or with broad immunoregulators (IMiDs), or chemotherapies with distinct mechanisms of action, remain the most promising strategies due to complex bi- or multi-directional interactions between MM cells with heterogenous genetic backgrounds and different accessory cells via multiple regulatory levels of ligand–receptor engagements in the BM microenvironment. Moreover, using more comprehensive next-generation proteomic and transcriptomic analysis from data longitudinally collected from patients in ongoing trials, novel druggable molecules will be discovered and new subsets of immune cells with more potent activity in regulating intrinsic and/or acquired immune resistance will be further identified.

## Figures and Tables

**Table 1:** Selected clinical trials of combination therapy or multi-target immunotherapy.

Clinical Trials	Agents	Format	Status
Anti-CD19/BCMA Bispecific CAR-T Cell Therapy for R/R MM (Phase 1, NCT03706547)	BCMA/CD19 CAR-T	CAR-T	Active, not recruiting
Anti-BCMA or/and Anti-CD19 CART Cells Treatment of Relapsed Multiple Myeloma (Phase 1, NCT03767725)	BCMA/CD19 CAR-T	CAR-T	Recruiting
Targeting CD19 and BCMA CAR-T Cells Immunotherapy in Patients with Relapsed or Refractory Multiple Myeloma (Phase 1+2, NCT04714827)	BCMA/CD19 CAR-T	CAR-T	Recruiting
A New Study Evaluating the Activity of Modular CAR T for mYeloma (MCARTY) (Phase 1, NCT04795882)	BCMA/CD19 CAR-T	CAR-T	Recruiting
A Feasibility and Safety Study of Dual Specificity CD38 and BCMA CAR-T Cell Immunotherapy for Relapsed or Refractory Multiple Myeloma (Phase 1+2, NCT03767751)	BCMA/CD38 CAR-T	CAR-T	Recruiting
BCMA-CS1 Compound CAR (cCAR) T Cells for Relapsed/Refractory Multiple Myeloma (Phase 1, NCT04156269)	BCMA-CS1 cCAR T	CAR-T	Recruiting
Safety and Efficiency Study of BCMA-PD1-CART Cells in Relapsed/Refractory Multiple Myeloma (Phase 2, NCT04162119)	BCMA-PD1-CART	CAR-T	Recruiting
BCMA-CD19 cCAR in Multiple Myeloma and Plasmacytoid Lymphoma (Phase 1, NCT04162353)	BCMA-CD19 cCAR	CAR-T	Recruiting
A Study of BCMA/CD19 Dual-Target CAR-T Cell Immunotherapy for Relapsed or Refractory Multiple Myeloma (Phase 1, NCT04182581)	BCMA/CD19 CAR-T	CAR-T	Recruiting
Humanized CAR-T Cells of Anti-BCAM and Anti-CD19 Against Relapsed and Refractory Multiple Myeloma (Phase 1, NCT04194931)	BCMA/CD19 CAR-T	CAR-T	Recruiting
A Study of BCMA/CD19 Dual-Target CAR-T Cell Immunotherapy for Relapsed or Refractory MM (Phase 1, NCT04412889)	BCMA/CD19 CAR-T	CAR-T	Recruiting
Up-front CART-BCMA with or without huCART19 in High-risk Multiple Myeloma (Phase 1, NCT03549442)	CART-BCMA, huCART19	CAR-T	Recruiting
Study of T Cells Targeting CD19/BCMA (CART-19/BCMA) for High-Risk Multiple Myeloma Followed with Auto-HSCT (Phase 1+2, NCT03455972)	BCMA/CD19 CAR-T	CAR-T	Recruiting
CAR-T Cells Combined with Dasatinib for Patients with Relapsed and/or Refractory B-cell Hematological Malignancies (Phase 1, NCT04603872)	BCMA/CD19 CAR-T	CAR-T	Recruiting
A Feasibility and Safety Study of Dual Specificity CD38 and BCMA CAR-T Cell Immunotherapy for Relapsed or Refractory Multiple Myeloma (Phase 1/2, NCT03767751)	CD38/BCMA CAR-T	CAR-T	Recruiting
BCMA-CS1 Compound CAR (cCAR) T Cells for Relapsed/Refractory Multiple Myeloma (Phase 1, NCT04156269)	BCMA-CS1 CAR-T	CAR-T	Recruiting
Study of T Cells Targeting CD138/BCMA/CD19/More Antigens (CART-138/BCMA/19/More) for Chemotherapy Refractory and Relapsed Multiple Myeloma (Phase 1, NCT03196414)	Multiple targets	CAR-T	Recruiting

The status of each trial is based on the description on the Clinicaltrials.gov website by 20 October 2021.
